# MWCNTs-TiO_2_ Incorporated-Mg Composites to Improve the Mechanical, Corrosion and Biological Characteristics for Use in Biomedical Fields

**DOI:** 10.3390/ma16051919

**Published:** 2023-02-25

**Authors:** Mohammad Taher Amirzade-Iranaq, Mahdi Omidi, Hamid Reza Bakhsheshi-Rad, Abbas Saberi, Somayeh Abazari, Nadia Teymouri, Farid Naeimi, Claudia Sergi, Ahmad Fauzi Ismail, Safian Sharif, Filippo Berto

**Affiliations:** 1 Advanced Materials Research Center, Department of Materials Engineering, Najafabad Branch, Islamic Azad University, Najafabad, Iran; t_amirzade@yahoo.com (M.T.A.-I.); abbassaberi65@gmail.com (A.S.); nadiateymouri7185@gmail.com (N.T.); farid.naeimi@gmail.com (F.N.); 2 Department of Materials and Metallurgical Engineering, Amirkabir University of Technology, Tehran, Iran; somayeh.abazari@gmail.com; 3 Department of Chemical Engineering Materials Environment, Sapienza University of Rome, Eudossiana 18, 00184 Roma, Italy; claudia.sergi@uniroma1.it; 4 Advanced Membrane Technology Research Center (AMTEC), Universiti Teknologi Malaysia, Johor Bahru 81310, Johor, Malaysia; afauzi@utm.my; 5 Advanced Manufacturing Research Group, Faculty of Mechanical Engineering, Universiti Teknologi Malaysia, Johor Bahru 81310, Johor, Malaysia; safian@utm.my

**Keywords:** magnesium matrix composites, TiO_2_-CNTs fillers, microstructure, mechanical property, biological behavior, corrosion property

## Abstract

This study attempts to synthesize MgZn/TiO_2_-MWCNTs composites with varying TiO_2_-MWCNT concentrations using mechanical alloying and a semi-powder metallurgy process coupled with spark plasma sintering. It also aims to investigate the mechanical, corrosion, and antibacterial properties of these composites. When compared to the MgZn composite, the microhardness and compressive strength of the MgZn/TiO_2_-MWCNTs composites were enhanced to 79 HV and 269 MPa, respectively. The results of cell culture and viability experiments revealed that incorporating TiO_2_-MWCNTs increased osteoblast proliferation and attachment and enhanced the biocompatibility of the TiO_2_-MWCNTs nanocomposite. It was observed that the corrosion resistance of the Mg-based composite was improved and the corrosion rate was reduced to about 2.1 mm/y with the addition of 10 wt% TiO_2_-1 wt% MWCNTs. In vitro testing for up to 14 days revealed a reduced degradation rate following the incorporation of TiO_2_-MWCNTs reinforcement into a MgZn matrix alloy. Antibacterial evaluations revealed that the composite had antibacterial activity, with an inhibition zone of 3.7 mm against *Staphylococcus aureus*. The MgZn/TiO_2_-MWCNTs composite structure has great potential for use in orthopedic fracture fixation devices.

## 1. **Introduction**

Magnesium alloys have many advantages as lightweight structural materials, which include their low density, high stiffness and specific strength, and great recyclability and cytocompatibility [1,2,3]. In the body’s fluid surroundings, they can gradually degrade which means there is no requirement for further operation to eliminate implants [4,5]. Mg ions, the body’s fourth most common ions, have been shown to stimulate bone regeneration and reduce healing rates [6,7]. Furthermore, MgZn alloys are comparable to real bones in terms of density and Young’s modulus, which lowers stress-shielding influence and deters osteoporosis [8,9,10]. However, rapid degradation in body fluid, which causes local alkalinity and hydrogen evolution, has proven to be complicated and potentially increases the risk of infection and implant failure. This is characterized by a lack of mechanical integrity because of rapid deterioration, crack propagation, and failure when still in use [11,12,13]. The time it takes for an implant to dissolve completely is determined by the location of the implant, the age of patient, the bone regeneration rate, and the mechanical strength of the broken bone [14]. Prior to the mechanical and structural characteristics deteriorating in the body and losing their effectiveness [15,16], a safety period of at least three months is recommended to allow for cell adhesion and hard bone tissue regeneration [17,18,19]. Nevertheless, Mg-based composites have demonstrated poor antibacterial efficiency, resulting in implants being associated with infections and post-operative complications. Bone infection is primarily a destructive problem with significant economic and clinical consequences [17,18,19,20]. As a result, there is a pressing need to improve Mg alloys’ antibacterial performance [8,20,21].

Hence, the encapsulation of an antibacterial agent into Mg-based matrix materials is a favorable method to diminish the viability, attachment, and growth of microbes on the surface of implants [20,21]. Because of its limitations, pure Mg cannot be used in service conditions without the addition of alloying elements or additional reinforcements [19]. The most common technique for enhancing the degradation resistance and antimicrobial performance of Mg is alloying and composite fabrication with appropriate nanofillers. Carbon nanotubes (CNTs) have generated great interest as a nanofiller because of their Young’s modulus (1 TPa), strength (30 GPa) and ultra-high thermal conductivity as well as their antimicrobial performance toward a wide variety of micro-organisms [7,11]. Because of these properties, CNTs are one of the best additives for Mg-based nanocomposites [12,14,22,23,24,25,26,27]. For example, Nai et al. [28] developed an Mg composite reinforced with CNTs via powder metallurgy with an excellent tensile strength (237 MPa); a 39% improvement compared to monolithic Mg. According to Han et al. [29], the elastic modulus and compressive yield strength of AZ31 composite containing CNTs were both improved in comparison with the AZ31 alloy. In the same manner, it was exhibited [3,12] that the addition of CNTs to Mg/Al composites increases ductility and decreases overall tensile strength. Because CNTs are one-dimensional and easily coagulated, this decrease in strength could be attributed to agglomerate formation. As a result, researchers face a significant challenge in achieving the uniform dispersion of CNTs in the matrix. As a result, many researchers have attempted to solve this problem through traditional mechanical milling and melting procedures [7,10,11,12]. However, these techniques could not be used to create composites with a uniform distribution of CNTs. Furthermore, CNTs were damaged during the traditional milling and casting procedures, causing breakage. To address these two issues (agglomerate formation of CNTs and CNT breakage), the semi-powder metallurgy (SPM) method is used to prepare the composites. SPM was used to prepare composites with a uniform dispersion of CNTs within the Mg-based matrix while avoiding CNT agglomeration and breakage [3,12]. However, until now, research on CNTs as reinforcement has primarily concentrated on mechanical properties, with little emphasis on the biocompatibility of Mg alloys [1,30,31]. However, these additions may affect the biosafety of magnesium alloys used in biological applications [32]. TiO_2_-based biomaterials are widely used as bone substitutes because of their biological compatibility with the body, and nanoscale TiO_2_ improves implant integration with host tissue in orthopedic applications [32,33,34]. Furthermore, nano-TiO_2_ has been reported to be biocompatible due to hydroxyapatite (HAp) formation via Ti–OH groups. It is corrosion resistant, has a large specific surface area, is non-toxic, flexible, high in tensile strength, has a relatively lower cost and a stable colloidal suspension [32,33,34]. It is possible to improve in vitro bioactivity of bone-like apatite development ability in simulated body fluid (SBF) by incorporating TiO_2_ nanoparticles into the composite [33,34]. However, findings from a published study [34] show that there has been no attempt to investigate the influences of TiO_2_ nano addition on the compressive and tensile performance of monolithic Mg. Currently, little research has been conducted to develop a new strategy to increase the antibacterial and mechanical characteristics of Mg metal matrix composites prepared with SPM. The goal of this study is to explore the feasibility of boosting the mechanical and antibacterial characteristics of Mg matrix nanocomposites by incorporating a TiO_2_-CNTs nanosystem during the manufacturing process. The mechanical alloying and SPM process coupled with spark plasma sintering (SPS) was used to create Mg/TiO_2_-CNTs composites with various amounts of TiO_2_-CNTs fillers, and their mechanical, corrosion and biological responses were investigated. The microstructural and mechanical characteristics of nanocomposites were investigated in comparison to those of an Mg alloy without TiO_2_-CNTs fillers.

## 2. Experimental Procedures

### 2.1. Materials and Preparation

Mg (size < 100 µm with 99.5% purity), Zn (size < 3 µm with 99.9% purity), and TiO_2_ (size < 500 nm with 99.5% purity) powders were provided by Merck Co. (Darmstadt, Germany). The procedure was conducted by mechanical alloying using 120 mL steel containers which were rotated at 600 rpm under an Ar gas atmosphere. A mixture of balls (ball diameter = 10 and 20 mm, mass = 35 g) were loaded into the container. The powder for the Mg–6Zn samples (Zn powders, 6 wt%; and Mg powders, 94 wt%) weighed about 12 g, where the ball material was zirconia with an approximate 20:1 mass ratio of powder to balls. The powder was prepared for 25 h at room temperature.

**Figure 1:**
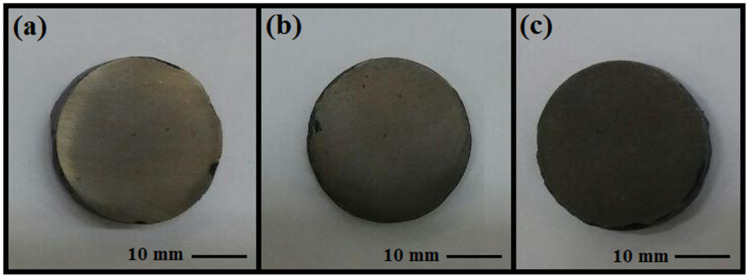
The prepared nanostructure (**a**) TM0, (**b**) TM1 and (**c**) TM3 composites fabricated via SPS methods.

To create Mg-6Zn/TiO_2_-MWCNTs composite via the SPM technique [32], TiO_2_ nanoparticles and CNTs as the reinforcement agents were mixed in a 10:1 weight ratio and were then denoted as TM0 (Mg-6Zn), TM1 (5TiO_2_-0.5MWCNTs), TM2 (10TiO_2_-1MWCNTs), and TM3 (15TiO_2_-1.5MWCNTs). In this regard, in order to achieve homogenous distribution, MgZn composite powder was stirred ultrasonically in 100% ethanol for 2 h. In this case, MgZn was used as a matrix and magnetic stirring was used to mix it for 1 h in ethanol at 600 rpm. At the same time, 0.5, 1, and 1.5 wt% MWCNTs and 5, 10, and 15 wt% TiO_2_ were ultrasonically dispersed for 3 h in ethanol. A solution containing magnesium composite and MWCNT was kept in the oven under controlled conditions for 1 day. The mixing procedure was carried for 3 h in order to obtain a homogenous mixture. To obtain the composite powder, the mechanically stirred mix was filtered and vacuum-dried at 70 °C overnight. The composite powders were sintered in the SPS chamber for 10 min at 570 °C and 40 MPa pressure to produce green billets with dimensions of 30 mm in diameter and 5 mm in thickness (Figure 1). 

### 2.2. Microstructure Characterization and Mechanical Property Testing

The composition distribution was examined via transmission electron microscopy (TEM; Phillips 208 m, (Amsterdam, The Netherlands)), field emission scanning electron microscope (FESEM, Tescan, Mira 3 Czech Republic, Prague, Czech Republic), and energy dispersive spectroscopy (JSM-5910LV, JOEL Ltd., Tokyo, Japan). Using an X-ray diffractometer, the constituent phases were identified (XRD, D8 Advance, Karlsruhe, Germany). The experimental parameters for XRD were Cu–Kα of X-ray and scan speed of 8° min^−1^. In order to assess the wettability of samples, the sessile drop method was used with a video contact angle device (GBX Digidrop, Romans-sur-Isère, France) and a drop size of 10 mL at room temperature. The results of 3 specimens were used to calculate the average contact angle for each kind of material. According to the ASTM-E9 standard, the compressive strength (SANTAM universal) of cylindrical composite materials with various contents of TiO_2_-MWCNTs was determined by pressing them at a weight of 10 kN and a speed of 2 mm/min. The microhardness of the samples was assessed using the Vickers microhardness (Shimadzu, Kyoto, Japan) test method with a load of 300 g.

### 2.3. Electrochemical and Immersion Tests

A potentiometric polarization experiment was carried out in the SBF using an EC-Lab device at a voltage range of between −250 and +250 mV_SCE_ and an open circuit potential at a rate of 0.5 mV/s in order to assess electrochemical behavior. The specimens were evaluated using a working electrode that had a surface area of 1 cm^2^ associated with exposure to the electrolyte, with a graphite electrode operating as the counter electrode and a saturated calomel electrode (SCE) acting as the reference electrode. The corrosion potential, corrosion current density, and slope of the anodic and cathode curves were also assessed using the EC-Lab express program. Electrochemical impedance spectrometry (EIS) was employed to establish potential stability following a 30 min sample immersion in the SBF. This test was performed in accordance with ASTM G106 standards utilizing a sign signal with a potential amplitude of 10 mV and an open circuit potential in the frequency band of 10^5^ to 10^−2^ Hz. The specimens were submerged in 200 mL of Kokubo SBF medium, in accordance with ASTM G1–03, for the immersion experiment. Throughout the immersion test, the pH change in SBF media was measured every 24 h.

The composites conducted mass loss testing, in accordance with the ASTM standard G31-72. During processing, the pH of the solution was measured using a pH meter. The volume of H_2_ gas emitted as a result of the Mg degradation was also measured, according to [20]. After the funneling and submerging of composites in SBF, a burette filled with SBF was positioned directly above them to collect the released H_2_ gas evolution and to measure its volume.

### 2.4. Antibacterial Properties

The biological properties of each Mg/TiO_2_-MWCNTs composite (*n* = 3) was examined, and the antibacterial activity against Gram-negative and Gram-positive bacteria was measured using the disc diffusion method. To accomplish this, lawns were used as the cultivation environments and sterile swabs dipped in the microbial suspension were flushed (by pressing swabs against a pipe’s side). The specimens were maintained at 37 °C for 1 day in an incubator while being incubated in an agar medium. If the specimens contain antibacterial capabilities, it will be visible in the inhibition area (IA) around them [35,36].

### 2.5. In Vitro Biocompatibility

The in vitro cytotoxicity of the Mg/TiO_2_-MWCNT composite was assessed using an indirect 3-(4,5-dimethylthiazol-2-yl)-2,5 diphenyl-tetrazolium-bromide (MTT, Sigma, Saint Louis, MO, USA) test according to the extraction method. The culture media was combined with the nanocomposites (5 mg) and incubated for 1 to 3 days at 37 °C. After 10^4^ cells/mL were placed on the 96-well plates for 24 h, the cell media were replenished with 1 and 3-day extracts. The medium was withdrawn after another 24 h, and 100 μL of MTT agents (0.5 mg/mL in PBS) were added to each well. The incubator was then left in place for 4 h. To dissolve formazan crystals, 100 μL of DMSO was then added into the well. The absorbance was then determined at 545 nm using an ELISA Reader (Stat Fax-2100, Miami, FL, USA) in comparison to a control group using free nanocomposites culture media. To investigate the alkaline phosphatase (ALP) activity of the samples, the composites were washed twice after culture for 3, and 7 days, according to [35]. The staining of ALP was performed using staining reagents. A microscope (TE2000U, Nikon, Tokyo, Japan) was then used to examine the stained cells [37].

### 2.6. Statistical Analysis

The test findings were provided as mean standard error (SE) and evaluated utilizing Sigmaplot software (version 12)** with (*) *p* < 0.05, (**) *p* < 0.01, and (***) *p* < 0.001 to reveal a considerable difference between data.

## 3. Results and Discussion

### 3.1. Microstructure Characterization, XRD and Raman Analysis

**Figure 2:**
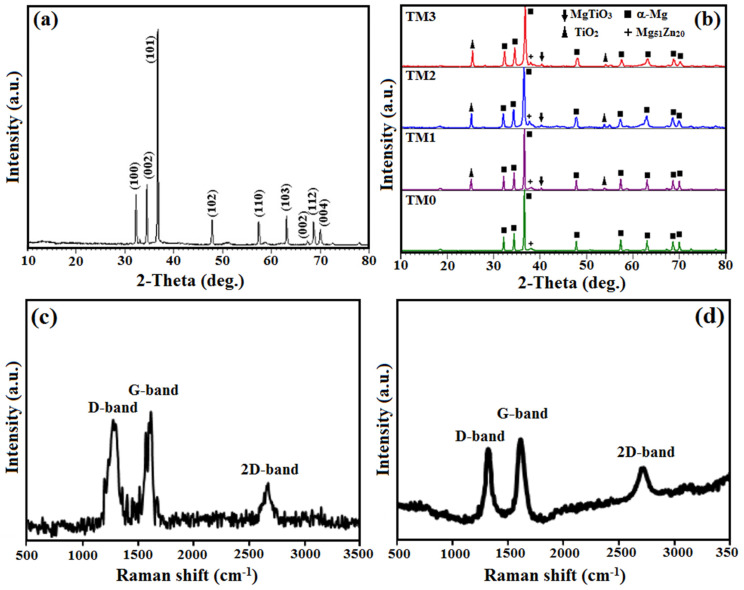
XRD pattern of (**a**) pure Mg and (**b**) TM0, TM1, TM2 and TM3 composites, (**c**) Raman spectra of MWCNTs and (**d**) TiO_2_-MWCNTs reinforced MgZn nanocomposite.

In Figure 2a, the peaks observed at 2θ = 32, 34, and 36° related to the (100) prism, (002) basal, and (101) pyramidal planes of HCP Mg structure, respectively [26,27]. The X-ray pattern of MgZn/TiO_2_-MWCNTs composites investigated along the cross-sections of the specimens is shown in Figure 2b. Meanwhile, the peak intensities corresponding to Mg_51_Zn_20_ in the Mg composite, and the existence of TiO_2_ and MgTiO_3_ peak suggests the integration of TiO_2_ in the Mg-based composite [38,39]. These precipitates act as barriers to grain boundary sliding, which limits further grain growth and thus improves the mechanical strength of the composite. The crystallite size of the composite was in the range of 64–87 nm, according to the Williamson–Hall equation [17]. The incorporation of TiO_2_ into the Mg-based composite caused a rise in reactive interface surfaces between the TiO_2_ and Mg powders via ball milling, facilitating the presence of reaction (1) through the applied mechanical self-propagating reaction (MSR) [40]. It is important to note that a small content of the geikielite (MgTiO_3_) phase forms in all milled specimens as a result of the following exothermic reaction [1]. Sul et al. [41] reported the cytocompatibility and bioresorbable quality of magnesium titanate oxide (MgTiO_3_) as a biological implant.

Mg + 1.5TiO_2_ ⟶ MgTiO_3_ + 0.5Ti (1)

Raman spectroscopy is commonly utilized to evaluate the structure of carbonaceous materials, crystallization, and imperfections. The Raman spectrum of the as-received MWCNTs revealed three different peaks: D (1340 cm^−1^), G (1585 cm^−1^) and 2D (2666 cm^−1^) (as shown in Figure 2c). The D peak resulted from structural defects and disorder in the carbon lattice, whereas the G peak resulted from sp^2^ bonding [42,43]. The two-phonon double resonance Raman phenomena were represented by the 2D peak. The I_D_/I_G_ ratio of the MWCNTs were enhanced from 0.82 to 0.97 in the MgZn/TiO_2_-MWCNTs composite powders (Figure 2d). As a result of the chemical oxidization and powder mixing operation, the degree of graphitization of the MWCNTs reduced, and the defects in the MWCNTs structure increased [42,43]. In this regard, it was demonstrated that [42] the acid oxidation reaction caused some minor etching of the MWCNTs surface, as well as continuous ball-to-powder contact during the ball milling procedure, which may have led to plastic deformation or fracture of the MWCNTs. This causes some structural damage to the MWCNTs. However, as compared to the G peak of the as-received MWCNTs, the G peak of the MgZn/TiO_2_-MWCNTs composite (1591 cm^−1^) shifted in the shorter wavelength direction. This peak shift was caused by the powder mixture procedure, which distorted the sp^2^ bonding microstructure of the MWCNTs. As a result, the blending operations of the introduced MWCNTs significantly affected the MWCNTs’ structure.

**Figure 3:**
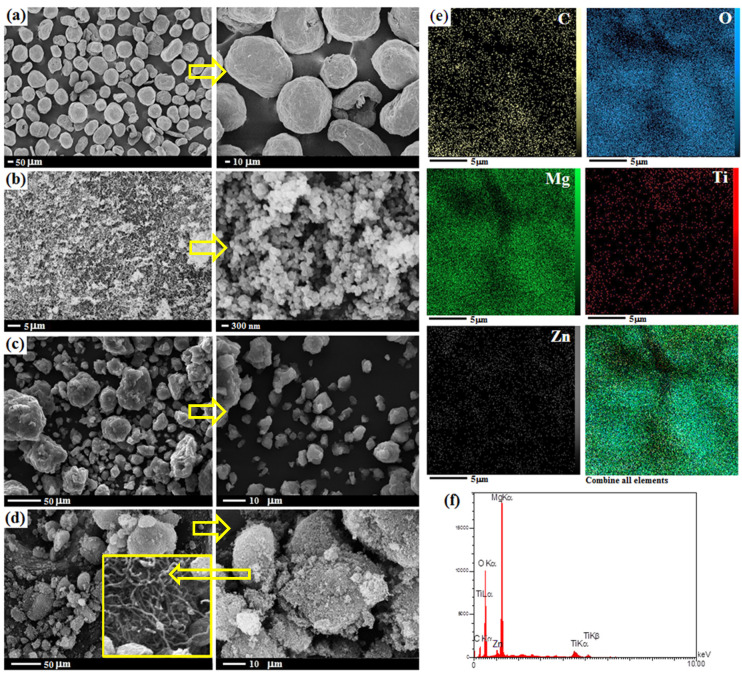
SEM images of (**a**) Mg, (**b**) TiO_2_, (**c**) MgZn/TiO_2_, and (**d**) TM2 composite and (**e**) EDS elemental mapping and (**f**) EDS analysis of TM2 powder composite.

SEM was used to examine the powder morphologies of pure Mg, nano-TiO_2_, MgZn/TiO_2_, and TM2 mixed powders, as shown in Figure 3. SEM images revealed that all Mg and TiO_2_ powders had spherical particles, but the nano-TiO_2_ powder had much finer particles (Figure 3a,b). Due to strong plastic deformation and the cold welding of Mg particles, milled Mg powders have a semi-spherical shape and a higher surface area. The efficient contact surfaces increase, making sintering densification easier. It is worth noting that it is preferable that the initial Mg and Zn powder particle sizes are quite different in order to achieve uniform Zn distribution in Mg. Because of the higher surface reactivity of Mg, it was complicated to utilize smaller sizes. On the other hand, the structure has Zn concentration of only 3 wt%. As a result, incorporating Zn particles of a similar size increases the risk of Zn concentration in specific areas of the composite structure. Furthermore, the homogeneous dispersion of Zn and TiO_2_ particles at the powder stage is aided by their smaller size and high surface area (Figure 3c). The aggregated morphology of MWCNTs is significantly tangled together. This microstructure might decrease the performance of MWCNTs as an additive. Fine particles of MgTiO_3_, TiO_2_ and MWCNTs are distributed and adhere homogeneously to the surface of Mg matrix alloy particles after the milling process, as shown in Figure 3d.

Details of the EDS elemental mapping data and SEM images show that the Zn element and nano-TiO_2_ reinforcements and MWCNTs were dispersed fairly on the Mg alloy surface powders, as shown in Figure 3e. SPM was utilized to fabricate Mg-based composites containing CNT and TiO_2_, where all powders were incorporated into the surface of a deformed Mg matrix during ball milling [44]. Different colors have been used to depict the cumulative and discrete dispersion of elements. Figure 3f depicts the elemental analysis (EDS) of the surface of Mg based alloy, revealing the existence of all the elements Zn, Ti, O, Mg and C in a homogeneous distribution [45]. The spectrum of the TM2 specimen contained Mg and Zn peaks, indicating that the powder was of high purity. The C peak in the EDX pattern can be verified by the incorporation of MWCNT on the synthesized Mg-based alloy specimen.

**Figure 4:**
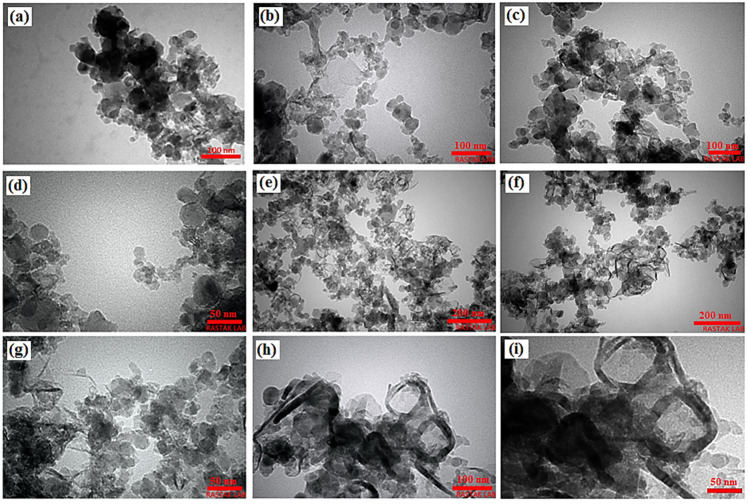
TEM images of (**a**) MgZn, (**b**–**d**) MgZn/TiO_2_, (**e**–**i**) MgZn/TiO_2_-MWCNTs (TM3) composite powders.

The morphological shape and size of the powder mixture of the MgZn, MgZn/TiO_2_, TiO_2_, MgZn/TiO_2_-MWCNTs (TM3) is exhibited by the TEM image in Figure 4. Milled MgZn powders have a spherical shape and increased surface area because of significant plastic deformation and the cold welding of Mg powders (Figure 4a), unlike the brittle components of MgZn/TiO_2_ that are attributed to a small particle size with various shapes (Figure 4b–d). The TM3 composite consisted of fine particles of MgTiO_3_, TiO_2_, and MWCNTs, where MWCNTs presented a diameter of 50 nm with homogeneous distribution and great adhesion within the matrix particle (Mg-based alloy), as shown in Figure 4e–i. Due to the fact that nearly all Mg particles were covered on the MWCNTs surface and only a few Mg powders remained outside the MWCNT support, the Mg-MWCNT/TiO_2_ interaction was observed to be strong.

**Figure 5:**
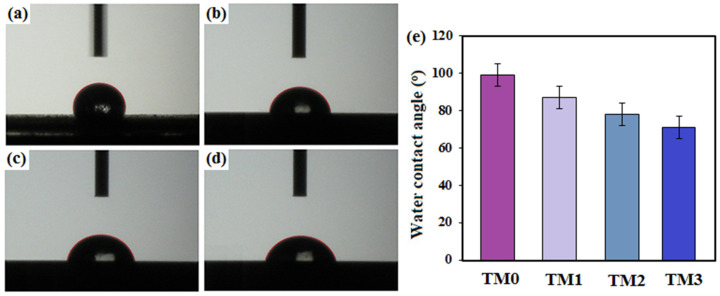
Contact angle of (**a**) TM0, (**b**) TM1, (**c**) TM2 and (**d**) TM3 composites and (**e**) values of the contact angles of the TM composites with various TiO_2_-MWCNTs content.

Wettability tests were also carried out using a contact angle instrument to determine the ability of the surfaces of the specimen to allow protein adhesion, which aids in cell attachment. The contact angles of the TM0, TM1, TM2 and TM3 composites are shown in Figure 5. The data from the in vitro cell culture test demonstrated that there is a significant relationship between the hydrophilicity of the specimen surfaces and cell behavior. Among all the samples, the TM0 composite had the highest hydrophilicity, with a contact angle of 99 ± 2.9°. The contact angles of the TM1 and TM2 samples were 87 ± 2.1° and 78 ± 1.3°, respectively [45]. The incorporation of MWCNTs enhanced the hydrophilic property of TM0 [41]. These results may be attributed to the hydrophilic nature of MWCNTs that contain a high concentration of hydroxyl functional groups [46]. It is well recognized that by increasing wettability the cell viability enhances. The MgZn base contains fewer voids and pores than the TM composites, which indicates that the corrosive medium penetrates the matrix of these composites less when compared to the TM3 containing 15 wt% TiO_2_ with contact angles of 72 ± 1.1°. As a result, the corrosion performance of the TM samples is highly influenced by the composite structure defect, with higher defects resulting in more corrosive solution infiltration and thus a lower corrosion rate.

### 3.2. Mechanical Characteristics

**Figure 6:**
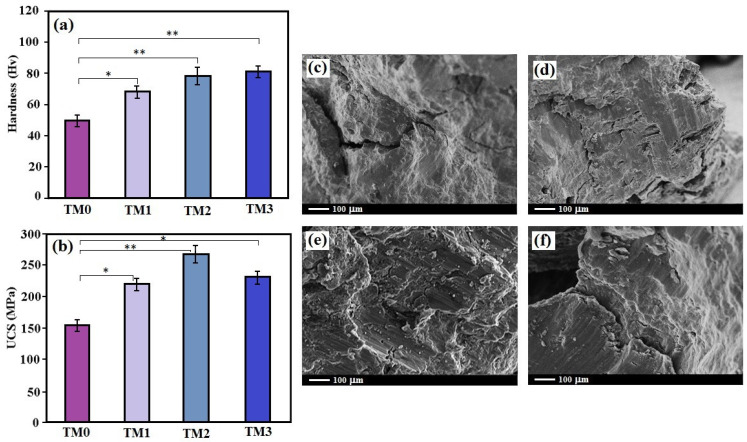
(**a**) The microhardness, (**b**) Ultimate compressive strength (UCS) of composites and SEM micrographs of fracture surfaces of (**c**) TM0, (**d**) TM1, (**e**) TM2 and (**f**) TM3 composites (* *p* < 0.05, and ** *p* < 0.01).

Figure 6a illustrates the microhardness experiments of the TM0, TM1, TM2 and TM3 nanocomposites. Compared to TM0, the incorporating TiO_2_-MWCNTs enhanced the hardness result considerably, with a maximum boost of approximately 64% observed with the incorporation of 10TiO_2_-1MWCNTs nanoparticles [34]. The hardness of the TM0, TM1, TM2 and TM3 composites was 50 ± 2.8 HV, 71 ± 3.3 HV, 79 ± 3.4 HV, and 75 ± 3.6 HV, respectively. The decrease in composite hardness for great incorporation of addition fractions (15TiO_2_-1.5CNTs) is attributed to the existence of agglomeration and, in particular, MWCNT segregation [47]. The main reason for the increase in hardness after the addition of 10TiO_2_-1CNTs fillers could be: (a) the appearance of TiO_2_, MgTiO_3_, and MgZn phases and MWCNTs fillers, (b) the presence of nanofillers that restrict localized deformation throughout indentation, and (c) grain size refinement [34].

Figure 6b shows the compression characteristics of the developed TM nanocomposite at room temperature. The ultimate compressive strength (UCS) of the TM1, TM2 and TM3 samples was greater than that of the TM0 sample. A maximum UCS (269 MPa) was detected for the TM2 sample, and a minimum UCS (152 MPa) was observed for the TM0. The boost in UCS of the nanocomposites may be related to: (a) grain size refinement, (b) Orowan strengthening attributed to the existence of nanofillers, (c) difference in the coefficient of thermal expansion values resulting in the formation of dislocations, and (d) the efficient load transfer from the matrix to MgTiO_3_, TiO_2_ and CNTs nanofillers. The capacity of a high-energy absorption up to failure under compression load, relating to the region under the stress–strain curve of the TM0 sample, was discovered to improve with the incorporation of the nanofillers. TM3 has a lower UCS than TM2 due to the formation of large agglomerates. Large, aggregated filler particles in the MgZn matrix would induce dislocation accumulation, causing crack formation and a lower compressive failure strain.

Figure 6c–f displays the fracture surfaces of the TM nanocomposite samples under the compression load. The fractographic images show that shear bands exist in all of the MgZn and nanocomposite specimens [34]. Several factors influence the reinforcing effectiveness of nanoscale additives such as MWCNTs fillers in the Mg matrix, including (1) the inherent mechanical characteristics of the reinforcement material, which includes TiO_2_ and MWCNTs, (2) the load transfer efficiency at the interface of the reinforcement and matrix, (3) the distribution level of the nanosize reinforcements in the Mg-based matrix, and (4) the uniform dispersion of the secondary phases and fillers in the Mg matrix [34]. Consolidation causes the MWCNTs to be bent or integrated between the Mg-based grains as a result of the force exerted on the MWCNTs by the matrix grains surrounding them, or if they are dispersed in the grain boundary and inside grain [48]. Because of the close contact between the MWCNTs and the grains, more binding occurs between the MWCNTs and the matrix grains, resulting in enhanced contact surface and mechanical interlocking, which results in improved load transfer performance between the Mg matrix and the MWCNTs. This has a significant effect on the mechanical characteristics of specimens [47]. These results demonstrate that uniform low contents of MWCNTs have a major influence on the mechanical characteristics of the sample.

Regarding the enhancing mechanism, mechanical alloying significantly reduces the second phase particle size in the Mg-based matrix from micron to nano length scale. Crack branching and load transfer are the toughening mechanisms that have been identified in all of the composites studied. The interaction of the propagating crack prevention and the TiO_2_-MWCNTs nano additions of varying sizes is the source of this mechanism. A crack is arrested and deflected in-plane when it propagates and comes in contact with the MgTiO_3_, TiO_2_, and MWCNTs nanofillers. It is well-known that such a crack deflection mechanism causes an extra tortuous route for stress release, where it enhances fracture toughness and the load transfer at the matrix-filler interface, which helps enhance the ductility and strength of Mg-based composites [47,48,49,50].

### 3.3. Corrosion Behavior

**Figure 7:**
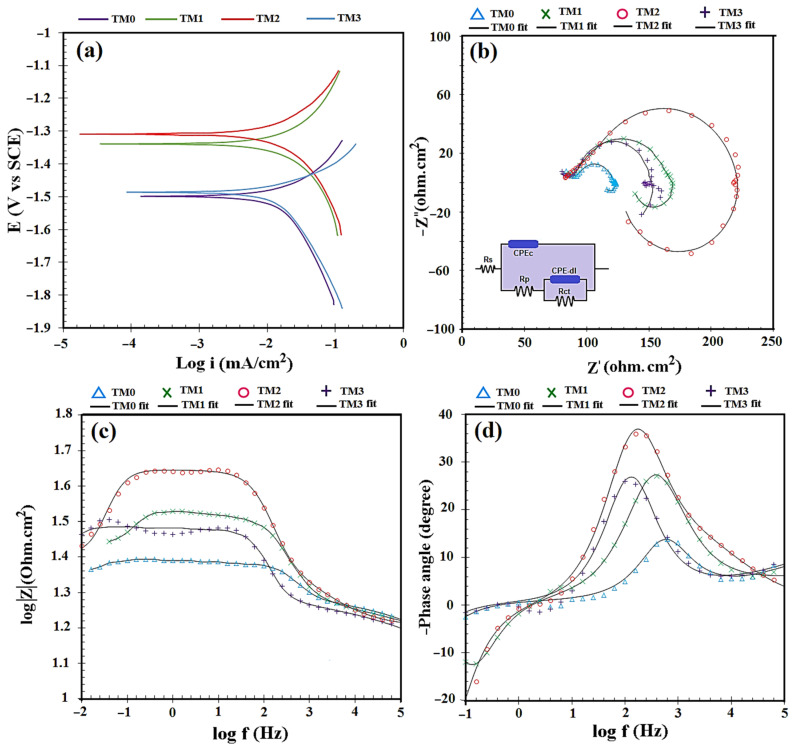
(**a**) Potentiodynamic polarization, (**b**) Nyquist plots, (**c**) Bode magnitude plot and (**d**) Bode phase curves of TM0, TM1, TM2 and TM3 composites.

To determine the corrosion resistance of the TM composites under test conditions, potentiodynamic polarization experiments were conducted. Figure 7a depicts the typical Tafel curves for potentiodynamic polarization tests on all composites. The addition of TiO_2_-MWCNTs reduces corrosion current density (I_corr_) and shifts corrosion potential (E_corr_) to the positive side. The electrochemical potential enhanced to −1498.9 mV_SCE_, which was considerably greater than the electrochemical potentials of TM2 (−1315.3 mV_SCE_), TM1 (−1338.7 mV_SCE_), and TM3 (−1480.8 mV_SCE_). A great reduction in current density and a significant boost in the electrochemical potential of polarization plots imply that the corrosion behavior of the TM composite has improved. Figure 7a presents the I_corr_ and E_corr_ values that correspond to the polarization curve. The low incorporation of TiO_2_-MWCNTs to Mg alloy noticeably reduced the I_corr_ of the composite. The addition of 15 TiO_2_-1.5 MWCNTs resulted in a slight decrease in the I_corr_. In this respect, the TM2 composite showed lower corrosion current density than the other composites in the following order: TM2 (118.6 µA/cm^2^) < TM1 (148.1 µA/cm^2^) < TM3 (156.4 µA/cm^2^) < TM0 (174.7 µA/cm^2^). This could be attributed to higher-content aggregation of 15 TiO_2_-1.5 CNTs, which function as just a secondary phase and cause more Mg degradation [11,51]. The existence of more fillers, including 15 TiO_2_-1.5 MWCNTs in the TM3 composite in comparison to TM2 and TM1, resulted in an increase in filler agglomerates. Furthermore, by adding more fillers (TM3 sample), the number of pores/voids around and/or within the TiO_2_-MWCNTs aggregates might be enhanced, resulting in a higher corrosion rate.

Figure 7b–d depicts the electrochemical impedance measured for the TM0, TM1, TM2 and TM3 composites. The diameter of the semicircles for the composites with a low filler content (TM1 and TM2) is clearly greater than that of the TM3 specimen, implying an escalation in corrosion resistance with the incorporation of a low number of fillers. The decrease in corrosion resistance of the specimen with high filler content (TM3), on the other hand, can be seen in the smaller diameter of the semicircles and defines the composite’s lower capacitive nature [15]. The equivalent circuit was comprised of a constant phase element (CPE), polarization resistance (Rp), charge transfer resistance (Rct) and solution resistance (Rs). The Rct value of TM1 (117 Ω·cm^2^) and TM2 (163 Ω·cm^2^) was greater than that of the TM3 (101 Ω·cm^2^) and TM3 (78 Ω·cm^2^) samples. It was shown that a composite containing a low content of TM1 and TM2 could greatly increase the corrosion resistance of the samples [52]. Similarly, the Bode magnitude and Bode phase curves exhibited the highest impedance modulus at a low-frequency (|Z|) and maximum phase angle for the TM1 and TM2 composites (Figure 7c,d). There are also reports [15,52] that a greater concentration of ceramic-based fillers led to more localized pitting corrosion of Mg-based composites. TiO_2_ and MWCNTs do not interact with metals throughout sintering due to their ceramic nature and high melting points, resulting in inadequate sintering at high TiO_2_ and MWCNTs content. This could also cause increased intensity of the galvanic couple, as TiO_2_ and MWCNTs are not well diffused in the matrix, resulting in a high corrosion rate, as seen in composite TM3. Furthermore, the corrosion rate was decreased by boosting the content and dispersion of TiO_2_ and MWCNTs [45]. It is important to note that the increase in corrosion resistance of the Mg-based composite containing ceramic additions can be related to a reduction in the number of pores and voids near the ceramic aggregates, which is induced by decreasing the ceramic content (TiO_2_-MWCNTs) of the Mg-based composite.

**Figure 8:**
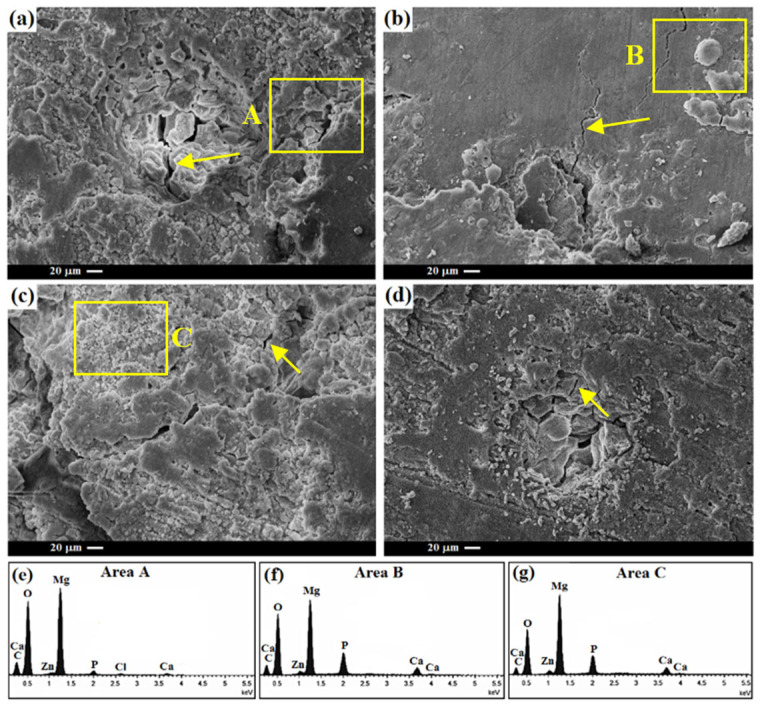
Surface morphology of the (**a**) TM0, (**b**) TM1, (**c**) TM2, (**d**) TM3 composites after 14 days of immersion in SBF and EDS analyses of (**e**) Area A, (**f**) Area B, and (**g**) Area C. Note: the arrow indicates the crack.

Figure 8a–d shows SEM images of the TM0, TM1, TM2 and TM3 samples after 14 days of immersion. On the surface of the TM0 sample, cracks (with an arrow) can be seen, which led to further penetration of the corrosive media and subsequently an escalation of the corrosion rate of the specimens during corrosion. The TM1 and TM2 composites hae fewer surface cracks than the TM3 composite with high TiO_2_-MWCNTs content. The TM2 specimen has the fewest corrosion cracks, which proves that the composite has good corrosion resistance and agrees completely with the outcomes of the polarization and EIS tests. On the corroded surface of the TM1 and TM2 biocomposites, there were fewer corrosion cracks and corrosion products (mainly white particles were visible); whereas, the TM3 composite had a severely corroded surface and corrosion products appear to cover the surface layer.

EDS analysis of these particles reveals that the product layer in point A-C was comprised of Mg, Ti, Ca, O and P elements, implying the possible development of apatite and Mg(OH)_2_ (Figure 8e–g). The corrosion products have a Ca/P ratio of 1.50, that is smaller than that of pure HA (Ca/P = 1.67) but close to that of calcium-phosphate (Ca/P = 1.63), implying that TiO_2_ and CNTs can form apatite. As a result of the greater calcium ion concentration in the SBF medium, greater negative charge, and many more nucleation sites, a TiO_2_-MWCNTs sample can create an apatite layer almost covering the entire composite surface, whereas apatite segments appear on the surface of the TM0 sample. Regarding formation of bone-like apatite, the apatite layer tends to attract phosphate ions in SBF, causing the development of a bone-like apatite cluster [53]. Because MWCNT reinforcements may influence both HA crystal nucleation and growth, the incorporation of MWCNTs had an influence on HA morphological changes. More specifically, the negatively charged carboxyl groups of MWCNTs could also function as a nucleation agent, which can attract Ca^2+^ ions and cause the deposition of HPO_4_^2−^ or PO_4_^3−^, finally resulting in the creation of HA [54]. 

**Figure 9:**
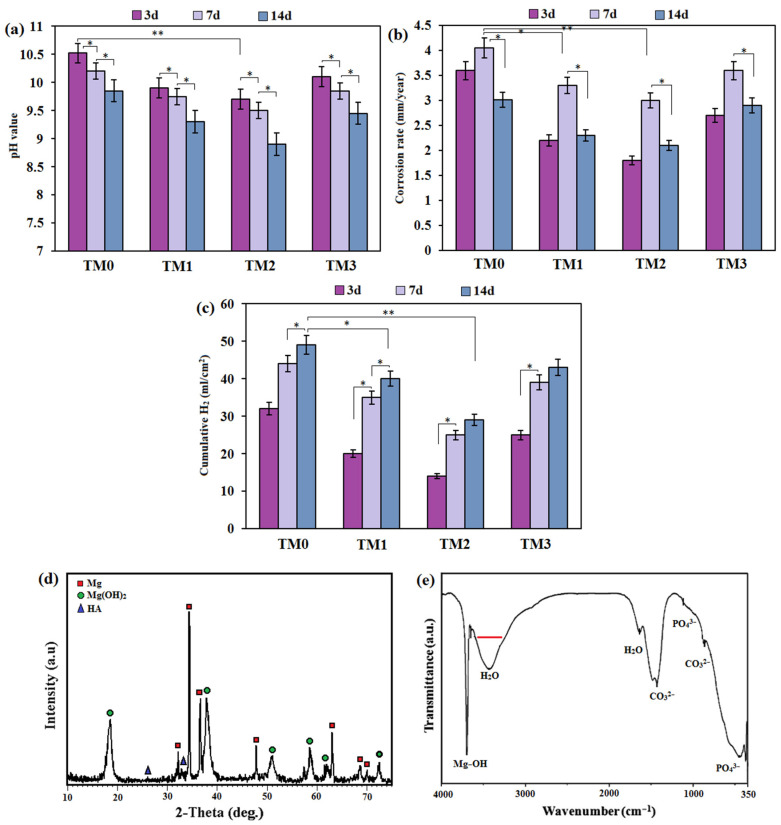
(**a**) pH value, (**b**) corrosion rate obtained by weight loss and (**c**) the rate of H_2_ evolution of TM0, TM1, TM2, and TM3 composites, (**d**) XRD pattern and (**e**) FTIR spectra of TM2 sample after 14 days of immersion in SBF (* *p* < 0.05, and ** *p* < 0.01).

Immersion experiments were carried out to determine their degradation properties further. The pH values observed in the experiment drastically enhanced during the immersion time in the first 48 h. On the other hand, pH showed a comparatively gentle, greatly increased trend (Figure 9a). More notably, the pH plots indicate that the TM2 biocomposite had the lowest pH value of 8.9, compared to 9.3 for TM1 and 9.4 for TM3 and 9.8 for TM0 biocomposites after 14 days of immersion in SBF.

Furthermore, the degradation rates from the weight loss investigation were efficiently calculated. The degradation rate of the TM0 sample (3.1 mm/y) was gradually reduced for TM2 (2.1 mm/y) and TM1 (2.4 mm/y), but greatly expanded for TM3 (2.9 mm/y) after 14 days of immersion in SBF (Figure 9b), indicating a relatively better degradation resistance [55]. The slope of the pH plot and the weight loss rate of the TM composite reduced because the immersion time was enhanced for all specimens. This result could be because of the decreased surface area of composite specimens, which allows the corrosion reaction to occur. Within the first few days of immersion, a larger surface area was exposed to solution leading to more degradation reactions and, as a result, an increase in the weight loss rate. The weight loss rate of the composite specimens lowered and then stabilized as the exposure time was extended to 7 and 14 days. This condition might be explained by the greater immersion time, which causes the creation of barrier layer on the surface of the composite specimens from corrosion products [49]. These films keep the samples from coming into direct contact with the solution, which reduces the weight loss rate of the composite samples. Figure 9c depicts the H_2_ gas evolution results for the TM composite, and the trend in the quantity of H_2_ gas evolved per unit surface area of the corrosion medium is nearly identical to that seen in the mass loss data. The TM1 specimen released almost 40.1 mL·cm^2^ in 14 days, compared to 49.2 mL·cm^2^ from the TM0 specimen. The amount of H_2_ gas evolved from the TM2 and TM3 specimens was 38.4 and 43.2 mL·cm^2^, respectively, after 14 days of immersion in SBF. The changes in mass loss and H_2_ evolution can be described using SEM images of the composites, which show that the protection provided by the TM1 and TM2 composites caused lower corrosion rates than TM0 [15]. These results suggest that the TM2 composite performs better than other composites in terms of degradation resistance. In this context, significant differences between corrosion rates of Mg-based composite without TiO_2_-MWCNTs (TM0) and with TiO_2_-MWCNTs (TM2) were observed (*p* < 0.05). However, there was no significant difference between the corrosion rate of TM1 and TM2 (*p* > 0.05) at a similar immersion time. Similarly, no significant differences (*p* > 0.05) were found between TM1 and TM2 for H_2_ evolution, but a significant difference between the H_2_ evolution of TM0 and TM2 (*p* < 0.05) was detected.

The XRD data of the specimens after 14 days of immersion is also shown in Figure 9d. The new peaks of the Mg(OH)_2_ and HAp precipitates could be seen for the TM2 composites when compared to the specimens without immersion, verifying the mineralization of the Mg matrix composites. The newly formed Mg(OH)_2_ and HAp precipitates sealed the pitting and cracks, allowing the Mg alloys to remain protected during immersion [39]. Biomineralized HAp precipitates a layer which is of great importance in influencing Mg corrosion due to its potentially dual role as a precipitate layer which involves: (1) influencing the kinetics of Mg resorption; and (2) affecting cell signaling for good tissue repair [39]. The FT-IR spectrum of the TM2 sample after 14 days of immersion in SBF is shown in Figure 9e. The triply devolved asymmetric stretching mode of the υ3PO_4_^3−^ bands at 1032 cm^−1^ and the υ4PO_4_^3−^ bands at 602 cm^−1^ and 565 cm^−1^ in the spectrum obviously demonstrated the presence of PO_4_^3−^ [55,56,57]. The spectrum exhibited CO_3_^2−^ absorption bands at approximately the same time, including the stretching mode of the υ1CO_3_^2−^ group in carbonated apatite at 1422 cm^−1^ and the characteristic bending mode of the υ4CO_3_^2−^ in carbonated apatite at 877 cm^−1^, indicating that the calcium phosphate development on the surface of the coated sample was comparable to the bone-like apatite. The band at 3704 cm^−1^ corresponds to Mg(OH)_2_ (brucite) in the FT-IR spectrum, and the OH group can be seen at 3416 cm^−1^ and 1624 cm^−1^ [56]. The adsorbed water was responsible for the broad absorption bands at 1635 cm^−1^.

### 3.4. Evaluation of Biocompatibility

**Figure 10:**
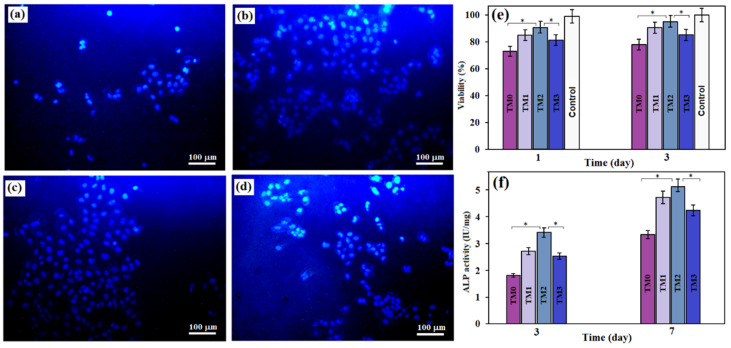
Fluorescent microscopic images of (**a**) TM0, (**b**) TM1, (**c**) TM2 and (**d**) TM3 (**e**) Cell viability and (**f**) ALP activity of TM0, TM1, TM2 and TM3 samples (* *p* < 0.05).

An MTT assay is a typical technique used to determine cell viability through a chemical reagent response. Figure 10a–d shows the fluorescence microscope image of the cell. Cell nuclei dyed with DAPI can be noticed as blue spots under a fluorescent microscope. Each spot represents the core of a single cell. It is clear that composites with low TiO_2_-MWCNTs amounts have greater total cell numbers than TM3 composites. The MTT assay shows that when osteoblast cells were co-cultured with TiO_2_-MWCNTs, cell viability increased with increasing time, implying that TiO_2_-MWCNTs affected cell viability. The TM1 and TM2 surfaces have shown a 92% and 96% increase in osteoblast cell viability, respectively, which is a greater increase than in the TM0 composite (80%) after 3 days of cultivation, as shown in Figure 10e. This suggests that TM2 is more suitable for cell adhesion than TM0 and TM3. There are numerous agglomerates on the composite surface of TM3, which may decrease cell adhesion. Interestingly, there is a significant difference between TM0 and TM2 for cell viability (*p* < 0.05). The number of cells increases as the amount of TiO_2_-MWCNTs increase up to a certain value (10TiO_2_-1MWCNTs), revealing that the surface structure of the specimen containing a low content of MWCNTs (0.5–1 wt%) is better for cell adhesion, whereas a higher amount of MWCNTs shows a lower number of cells, indicating cytocompatibility of the TM specimens with lower concentrations of MWCNTs (up to 1 wt%). The lower pH and H_2_ release in the TM1 and TM2 biocomposite extracts may illustrate the higher cell viability of these composites compared to the TM0 and TM3. These findings showed that integrating a small content of TiO_2_-MWCNTs improved the biocompatibility of the TM0 biocomposite [39,45,47,55]. A lower content of CNT (≈5 vol%) has been recommended in the composite in order to control the amount entering the human body [11]. The biosafety of these particles is ensured by the immobilization of CNT in metallic biomaterials, which prevents their direct exposure to surrounding tissues [7,11]. However, further research is needed to determine their safe clinical use as novel biomaterials for use in tissue engineering as CNT is safe when applied topically but not in certain areas such as the lungs and the abdominal cavity.

The ALP assay was used to evaluate the effect of material extracts on osteogenesis differentiation. During the first three days of incubation, the TM2 composite provided better stimulation for cell differentiation. However, when compared to TM0, the ALP activity of the TM3 composite is higher. It should be noted that increasing the incubation period to 5 days increased ALP activity in all groups, particularly in the TM1 and TM2 groups. This could be attributed to bone mineralization and implies that Mg-containing 10TiO_2_-1MWCNTs increased the ALP activity of cells grown on the surface. This is because, following the incorporation of TiO_2_-MWCNTs, a bioactive apatite layer precipitates on the composite surface, causing the binding of proteins and other bio-organic substances used to stimulate cell attachment and proliferation [57]. Cell viability was found to be less for TM3 compared to TM1 and TM2. At higher concentrations, the addition of 15TiO_2_-1.5MWCNTs tends to aggregate and act as a secondary phase in the sample, which increases the corrosion rate and hence reduces cell viability [58]. Cell adhesion and proliferation are physically inhibited by the corroded surface. Thus, a low content of MWCNTs (0.5–1 wt%) is proposed as the optimal content for boosting the biocompatibility of the composite. Evidently, there was no significant difference between the cell viability of TM1 and TM2 (*p* > 0.05) at a similar cultivation time. Webster et al. [59] discovered a boost in the number of adherent osteoblasts after incorporating carbon nanofibres into polycarbonate urethane. This is most likely the result of increased material deficiencies and electron delocalization on the nanostructured surface. Furthermore, the nanoscale topography of the TiO_2_ surface affects osteoblast functions such as attachment and proliferation, with broad nanosheets (>100 nm) being ideal for osteoblast attachment [27]. 

### 3.5. Antibacterial Properties

**Figure 11:**
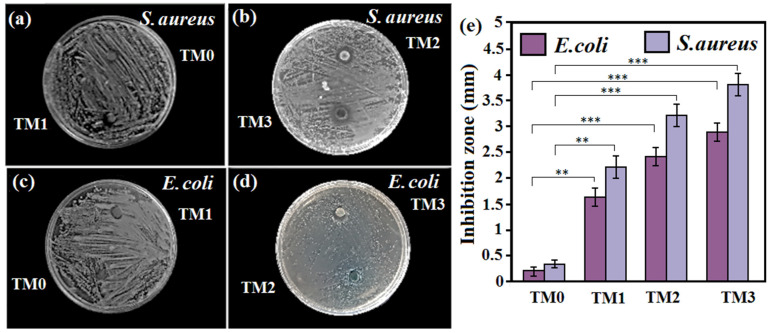
(**a**) Antibacterial activities of the (**a**) TM0 and TM1, (**b**) TM2 and TM3, (**c**) TM0 and TM1, and (**d**) TM2 and TM3 composites by the disc diffusion test against both Gram-positive (*S. aureus*) and Gram-negative (*E. coli*) and (**e**) diameters of the inhibition zones for TM0, TM1, TM2 and TM3 samples (** *p* < 0.01 and *** *p* < 0.001).

The antibacterial performance of the TM0, TM1, TM2 and TM3 composites was evaluated functionally using inhibition area (IA) for 24 h (Figure 11a–d). Figure 11 shows that as the concentration of TiO_2_-MWCNTs increased, subsequently the antibacterial activity of the composite escalated. Antibacterial activity was determined after 24 h of incubation by measuring the inhibition area diameter and is shown in Figure 11e. The inhibition zone of TM1 and TM2 was 2.2 mm and 3.3 mm, respectively, against *Staphylococcus aureus* (*S. aureus*) indicating effective antibacterial performance against Gram-positive bacteria. It was also discovered that incorporating fillers (TiO_2_-MWCNTs) and the boost in filler concentration resulted in a higher diameter zone against Gram-negative and Gram-positive bacteria in all cases. The bacteriostatic activity was high in the composite loaded with TM3, with a high inhibition zone of 3.7 mm against *S. aureus*. The disc diffusion tests show that the inhabitation area (IA) around the Mg-based composite without TiO_2_-MWCNTs (TM0) is significantly smaller (*p* < 0.001) than that of the composite containing TiO_2_-MWCNTs fillers. These findings demonstrate that the TiO_2_-MWCNTs-loaded composites developed in this research have strong antimicrobial activity, with the majority of bacteria being inhibited from growing or even killed. The findings revealed that the TM2 and TM3 composites containing 10–15 wt% TiO_2_ and 1–1.5 wt% MWCNTs fillers had increased activity against the bacterial species, namely, *S. aureus* and *Escherichia coli* (*E. coli*). According to the results in Figure 11e, the diameter zone of *S. aureus* bacteria was slightly greater than that of *E. coli* bacteria, which implies that there was no significant difference between the IA values (*p* > 0.05) in *E. coli* or *S. aureus* for any sample.

The obtained inhibition zone is attributable to the influence of the MWCNTs and TiO_2_ nano additions. Furthermore, the titanium ion release mechanism can improve antibacterial performance [60]. Moreover, because of regulatory and signal dysfunction, phosphorous and iron motion, and heme groups along the cell wall, TiO_2_NPs have antimicrobial activity. The antibacterial performance of TiO_2_ nano additions may also be due to the generation of reactive oxygen species and Ti ions [61,62,63]. Furthermore, several prior studies [64,65] have suggested that the antibacterial performance of CNTs is associated with cell membrane destruction induced by direct contact between agglomerated CNT and bacteria. Individually dispersed CNTs can be illustrated as numerous moving “nano darts” in the medium, which are continuously trying to attack the bacteria, thus also degrading the integrity of bacterial cell and ultimately leading to cell death, as demonstrated by Liu et al. [64]. Previous research has linked CNT antibacterial activity to the physical puncture of agglomerated CNT, leading to physical damage to the cell’s outer membrane. As a result, the well-dispersed surface with identifiable active particles and fillers leads to the inhibition of bacteria. Taken together, the addition of various amounts of fillers, such as TiO_2_ and CNTs, into the composites has a significant influence on the biological response, corrosion behavior and mechanical properties of the Mg-based composite [66,67,68,69,70,71,72,73,74,75,76,77,78,79,80,81,82,83].

## 4. Conclusions

This study used mechanical alloying and semi-powder metallurgy processes combined with spark plasma sintering to fabricate high-density MgZn/TiO_2_-MWCNTs composites. The MgZn matrix co-encapsulated with TiO_2_ and MWCNT nano additives has considerably higher strength than that required to meet the mechanical characteristics of biomedical devices. The incorporation of TiO_2_-MWCNTs fillers into the MgZn matrix largely controlled the adjustable mechanical characteristics and proper corrosion rate. The corrosion rate of MgZn/TiO_2_-MWCNTs composite (~2.1 mm/y) is lower than that of MgZn (~3 mm/y) when protected by a more even Ca-P layer with fewer cracks and defects on the surface of the composite. The MgZn/TiO_2_-MWCNTs composite is biocompatible with MG-63 cells in vitro, and the cytocompatibility of TM composite with low TiO_2_-MWCNTs content is slightly better than that of the MgZn composite. The findings also revealed that co-incorporating MWCNTs and TiO_2_ into the Mg-based matrix had a considerable effect on antibacterial action by physical puncture of CNT aggregates and more penetration of TiO_2_ nanoparticles, leading to physical damage of the outer membrane of cells with subsequent degradation of bacterial cell integrity and cell death. The TM1 and TM2 composites containing 5–10 wt% TiO_2_ and 0.5–1 wt% MWCNTs fillers may be potential candidates for use in biodegradable implants due to their excellent mechanical and antibacterial characteristics, acceptable biocompatibility, and relatively low in vitro corrosion rate.

## Data Availability

All data provided in the present manuscript are available to whom it may concern.

## References

[1] Hou J., Du W., Wang Z., Li S., Liu K., Du X. (2020). Combination of enhanced thermal conductivity and strength of MWCNTs reinforced Mg-6Zn matrix composite. J. Alloys Compd..

[2] Shuai C., Liu L., Zhao M., Feng P., Yang Y., Guo W., Gao C., Yuan F. (2018). Microstructure, biodegradation, antibacterial and mechanical properties of ZK60-Cu alloys prepared by selective laser melting technique. J. Mater. Sci. Technol..

[3] Rashad M., Pan F., Tang A., Lu Y., Asif M., Hussain S., She J., Gou J., Mao J. (2013). Effect of graphene nanoplatelets (GNPs) addition on strength and ductility of magnesium-titanium alloys. J. Magnes. Alloys.

[4] Mindivan H., Efe A., Kosatepe A.H., Kayali E. (2014). Fabrication and characterization of carbon nanotube reinforced magnesium matrix composites. Appl. Surf. Sci..

[5] Xiong P., Jia Z., Li M., Zhou W., Yan J., Wu Y., Cheng Y., Zheng Y. (2018). Biomimetic Ca, Sr/P-Doped Silk Fibroin Films on Mg-1Ca Alloy with Dramatic Corrosion Resistance and Osteogenic Activities. ACS Biomater. Sci. Eng..

[6] Shuai C., Wang B., Yang Y., Peng S., Gao C. (2019). 3D honeycomb nanostructure-encapsulated magnesium alloys with superior corrosion resistance and mechanical properties. Compos. Part B Eng..

[7] Abazari S., Shamsipur A., Bakhsheshi-Rad H.R., Ismail A.F., Sharif S., Razzaghi M., Ramakrishna S., Berto F. (2020). Carbon Nanotubes (CNTs)-Reinforced Magnesium-Based Matrix Composites: A Comprehensive Review. Materials.

[8] Chen Y., Zhang S., Li J., Song Y., Zhao C., Zhang X. (2010). Dynamic degradation behavior of MgZn alloy in circulating m-SBF. Mater. Lett..

[9] Zhou J., Zhong K., Zhao C., Meng H., Qi L. (2021). Effect of carbon nanotubes grown temperature on the fracture behavior of carbon fiber reinforced magnesium matrix composites: Interlaminar shear strength and tensile strength. Ceram. Int..

[10] Shen M., Han B., Ying T. (2022). Micromechanical simulations and experimental characteristics of randomly distributed carbon nanotubes reinforced Mg matrix composites. J. Alloys Compd..

[11] Munir K.S., Wen C., Li Y.C. (2019). Carbon Nanotubes and Graphene as Nanoreinforcements in Metallic Biomaterials: A Review. Adv. Biosyst..

[12] Rashad M., Pan F., Tang A., Asif M., Hussain S., Gou J., Mao J. (2015). Improved strength and ductility of magnesium with addition of aluminum and graphene nanoplatelets (Al+GNPs) using semi powder metallurgy method. J. Ind. Eng. Chem..

[13] Li L.-Y., Cui L.-Y., Zeng R.-C., Li S.-Q., Chen X.-B., Zheng Y., Kannan M.B. (2018). Advances in functionalized polymer coatings on biodegradable magnesium alloys—A review. Acta Biomater..

[14] Xiang Y., Wang X., Hu X., Meng L., Song Z., Li X., Sun Z., Zhang Q., Wu K. (2019). Achieving ultra-high strengthening and toughening efficiency in carbon nanotubes/magnesium composites via constructing micro-nano layered structure. Compos. Part A Appl. Sci. Manuf..

[15] Prabhu D.B., Gopalakrishnan P., Ravi K. (2020). Morphological studies on the development of chemical conversion coating on surface of Mg–4Zn alloy and its corrosion and bio mineralisation behaviour in simulated body fluid. J. Alloys Compd..

[16] Sezer N., Evis Z., Koç M. (2021). Additive manufacturing of biodegradable magnesium implants and scaffolds: Review of the recent advances and research trends. J. Magnes. Alloys.

[17] Li Y., Zhou J., Pavanram P., Leeflang M.A., Fockaert L.I., Pouran B., Zadpoor A.A. (2018). Additively manufactured biodegradable porous magnesium. Acta Biomater..

[18] Xing F., Li S., Yin D., Xie J., Rommens P.M., Xiang Z., Liu M., Ritz U. (2022). Recent progress in Mg-based alloys as a novel bioabsorbable biomaterials for orthopedic applications. J. Magnes. Alloys.

[19] Hou J., Du W., Parande G., Gupta M., Li S. (2019). Significantly enhancing the strength + ductility combination of Mg-9Al alloy using multi-walled carbon nanotubes. J. Alloys Compd..

[20] Song J., Chen J., Xiong X., Peng X., Chen D., Pan F. (2022). Research advances of magnesium and magnesium alloys worldwide in 2021. J. Magnes. Alloys.

[21] Nie K., Wang X., Deng K., Hu X., Wu K. (2021). Magnesium matrix composite reinforced by nanoparticles—A review. J. Magnes. Alloys.

[22] Esmaily M., Svensson J.E., Fajardo S., Birbilis N., Frankel G.S., Virtanen S., Arrabal R., Thomas S., Johansson L.G. (2017). Fundamentals and advances in magnesium alloy corrosion. Prog. Mater. Sci..

[23] Zheng Y.F., Gu X.N., Witte F. (2014). Biodegradable metals. Materials Science and Engineering: R: Reports.

[24] Yuan Q.-H., Zeng X.-S., Liu Y., Luo L., Wu J.-B., Wang Y.-C., Zhou G.-H. (2016). Microstructure and mechanical properties of AZ91 alloy reinforced by carbon nanotubes coated with MgO. Carbon.

[25] Li X., Liu X., Wu S., Yeung K., Zheng Y., Chu P.K. (2016). Design of magnesium alloys with controllable degradation for biomedical implants: From bulk to surface. Acta Biomater..

[26] Kaushik V., Kumar B.N., Kumar S.S., Vignesh M. (2022). Magnesium role in additive manufacturing of biomedical implants–challenges and opportunities. Addit. Manuf..

[27] Yuan Q.-H., Zhou G.-H., Liao L., Liu Y., Luo L. (2018). Interfacial structure in AZ91 alloy composites reinforced by graphene nanosheets. Carbon.

[28] Nai M.H., Wei J., Gupta M. (2014). Interface tailoring to enhance mechanical properties of carbon nanotube reinforced magnesium composites. Mater. Des..

[29] Han G., Wang Z., Liu K., Li S., Du X., Du W. (2015). Synthesis of CNT-reinforced AZ31 magnesium alloy composites with uniformly distributed CNTs. Mater. Sci. Eng. A.

[30] Ali M., Hussein M., Al-Aqeeli N. (2019). Magnesium-based composites and alloys for medical applications: A review of mechanical and corrosion properties. J. Alloys Compd..

[31] Paramsothy M., Chan J., Kwok R., Gupta M. (2011). Addition of CNTs to enhance tensile/compressive response of magnesium alloy ZK60A. Compos. Part A Appl. Sci. Manuf..

[32] Shanmugam B.K., Rangaraj S., Subramani K., Srinivasan S., Aicher W.K., Venkatachalam R. (2020). Biomimetic TiO_2_-chitosan/sodium alginate blended nanocomposite scaffolds for tissue engineering applications. Mater. Sci. Eng. C.

[33] Menazea A., Awwad N.S. (2020). Antibacterial activity of TiO_2_ doped ZnO composite synthesized via laser ablation route for antimicrobial application. J. Mater. Res. Technol..

[34] Meenashisundaram G.K., Nai M.H., Almajid A., Gupta M. (2015). Development of high performance Mg–TiO_2_ nanocomposites targeting for biomedical/structural applications. Mater. Des..

[35] Bakhsheshi-Rad H.R., Ismail A.F., Aziz M., Akbari M., Hadisi Z., Khoshnava S.M., Pagan E., Chen X. (2020). Co-incorporation of graphene oxide/silver nanoparticle into poly-L-lactic acid fibrous: A route toward the development of cytocompatible and antibacterial coating layer on magnesium implants. Mater. Sci. Eng. C.

[36] Abazari S., Shamsipur A., Bakhsheshi-Rad H.R. (2021). Reduced graphene oxide (RGO) reinforced Mg biocomposites for use as orthopedic applications: Mechanical properties, cytocompatibility and antibacterial activity. J. Magnes. Alloys.

[37] Shuai C., Zan J., Qi F., Wang G., Liu Z., Yang Y., Peng S. (2019). nMgO-incorporated PLLA bone scaffolds: Enhanced crystallinity and neutralized acidic products. Mater. Des..

[38] Saberi A., Bakhsheshi-Rad H., Karamian E., Kasiri-Asgarani M., Ghomi H. (2020). Magnesium-graphene nano-platelet composites: Corrosion behavior, mechanical and biological properties. J. Alloys Compd..

[39] Zhang C., Ding Z., Xie L., Zhang L.-C., Wu L., Fu Y., Wang L., Lu W. (2017). Electrochemical and in vitro behavior of the nanosized composites of Ti-6Al-4V and TiO_2_ fabricated by friction stir process. Appl. Surf. Sci..

[40] Khalajabadi S.Z., Kadir M.R.A., Izman S., Bakhsheshi-Rad H.R., Farahany S. (2014). Effect of mechanical alloying on the phase evolution, microstructure and bio-corrosion properties of a Mg/HA/TiO_2_/MgO nanocomposite. Ceram. Int..

[41] Sul Y.-T. (2008). Osseoinductive Magnesium-Titanate Implant and Method of Manufacturing the Same. U.S. Patent.

[42] Ramezanzade S.G.R., Ebrahimi M. (2019). Torabi Parizi and H.R. Ezatpour, Synergetic effect of GNPs and MgOs on the mechanical properties of Mg–Sr–Ca alloy. Mater. Sci. Eng. A.

[43] Parizi M.T., Ebrahimi G., Ezatpour H., Paidar M. (2019). The structure effect of carbonaceous reinforcement on the microstructural characterization and mechanical behavior of AZ80 magnesium alloy. J. Alloys Compd..

[44] Cui Z., Li W., Cheng L., Gong D., Cheng W., Wang W. (2019). Effect of nano-HA content on the mechanical properties, degradation and biocompatible behavior of Mg-Zn/HA composite prepared by spark plasma sintering. Mater. Charact..

[45] Jaiswal S., Kumar R.M., Gupta P., Kumaraswamy M., Roy P., Lahiri D. (2018). Mechanical, corrosion and biocompatibility behaviour of Mg-3Zn-HA biodegradable composites for orthopaedic fixture accessories. J. Mech. Behav. Biomed. Mater..

[46] Shuai C., Zeng Z., Yang Y., Qi F., Peng S., Yang W., He C., Wang G., Qian G. (2020). Graphene oxide assists polyvinylidene fluoride scaffold to reconstruct electrical microenvironment of bone tissue. Mater. Des..

[47] Baradaran S., Moghaddam E., Basirun W.J., Mehrali M., Sookhakian M., Hamdi M., Moghaddam M.R.N., Alias Y. (2014). Mechanical properties and biomedical applications of a nanotube hydroxyapatite-reduced graphene oxide composite. Carbon.

[48] Shahin M., Munir K., Wen C., Li Y. (2020). Magnesium-based composites reinforced with graphene nanoplatelets as biodegradable implant materials. J. Alloys Compd..

[49] Shahin M., Munir K., Wen C., Li Y. (2019). Magnesium matrix nanocomposites for orthopedic applications: A review from mechanical, corrosion, and biological perspectives. Acta Biomater..

[50] Shahin M., Munir K., Wen M., Wen C., Li Y. (2021). Microstructure, mechanical and corrosion properties of hot-pressed graphene nanoplatelets-reinforced Mg matrix nanocomposites for biomedical applications. J. Alloys Compd..

[51] Sunil B.R., Ganapathy C., Kumar T.S., Chakkingal U. (2014). Processing and mechanical behavior of lamellar structured degradable magnesium–hydroxyapatite implants. J. Mech. Behav. Biomed. Mater..

[52] Chen Y., Wang J., Dou J., Yu H., Chen C. (2020). Layer by layer assembled chitosan (TiO_2_)-heparin composite coatings on MAO-coated Mg alloys. Mater. Lett..

[53] Zhang L., Liu W., Yue C., Zhang T., Li P., Xing Z., Chen Y. (2013). A tough graphene nanosheet/hydroxyapatite composite with improved in vitro biocompatibility. Carbon.

[54] Khazeni D., Saremi M., Soltani R. (2019). Development of HA-CNTs composite coating on AZ31 Magnesium alloy by cathodic electrodeposition. Part 2: Electrochemical and in-vitro behavior. Ceram. Int..

[55] Shuai C., Wang B., Bin S., Peng S., Gao C. (2020). TiO_2_-Induced In Situ Reaction in Graphene Oxide-Reinforced AZ61 Biocomposites to Enhance the Interfacial Bonding. ACS Appl. Mater. Interfaces.

[56] Wei D., Du Q., Guo S., Jia D., Wang Y., Li B., Zhou Y. (2018). Structures, bonding strength and in vitro bioactivity and cytotoxicity of electrochemically deposited bioactive nano-brushite coating/TiO_2_ nanotubes composited films on titanium. Surf. Coat. Technol..

[57] Singh S., Kumar R.M., Kuntal K.K., Gupta P., Das S., Jayaganthan R., Roy P., Lahiri D. (2015). Sol–Gel Derived Hydroxyapatite Coating on Mg-3Zn Alloy for Orthopedic Application. Jom.

[58] Willumeit R., Möhring A., Feyerabend F. (2014). Optimization of Cell Adhesion on Mg Based Implant Materials by Pre-Incubation under Cell Culture Conditions. Int. J. Mol. Sci..

[59] Webster T.J., Waid M.C., McKenzie J.L., Price R.L., Ejiofor J.U. (2004). Nano-biotechnology: Carbon nanofibres as improved neural and orthopaedic implants. Nanotechnology.

[60] Abutalib M., Rajeh A. (2021). Enhanced structural, electrical, mechanical properties and antibacterial activity of Cs/PEO doped mixed nanoparticles (Ag/TiO_2_) for food packaging applications. Polym. Test..

[61] Shi J., Li J., Wang Y., Zhang C.Y. (2022). TiO_2_-based nanosystem for cancer therapy and antimicrobial treatment: A review. Chem. Eng. J..

[62] Vargas M.A., Rodríguez-Páez J.E. (2017). Amorphous TiO_2_ nanoparticles: Synthesis and antibacterial capacity. J. Non-Crystalline Solids.

[63] Ulu A., Birhanlı E., Köytepe S., Ateş B. (2020). Chitosan/polypropylene glycol hydrogel composite film designed with TiO_2_ nanoparticles: A promising scaffold of biomedical applications. Int. J. Biol. Macromol..

[64] Liu S., Wei L., Hao L., Fang N., Chang M.W., Xu R., Yang Y., Chen Y. (2009). Sharper and Faster “Nano Darts” Kill More Bacteria: A Study of Antibacterial Activity of Individually Dispersed Pristine Single-Walled Carbon Nanotube. ACS Nano.

[65] Liu S., Zeng T.H., Hofmann M., Burcombe E., Wei J., Jiang R., Kong J., Chen Y. (2011). Antibacterial Activity of Graphite, Graphite Oxide, Graphene Oxide, and Reduced Graphene Oxide: Membrane and Oxidative Stress. ACS Nano.

[66] Weng Z., Zou F., Li D., Yao Y. (2022). A high-sensitivity thin-film MWNT@ PDA-AgNP nanocomposite sensor for acquiring microscopic deformations. Compos. Sci. Technol..

[67] Naebe M., Lin T., Tian W., Dai L., Wang X. (2007). Effects of MWNT nanofillers on structures and properties of PVA electrospun nanofibres. Nanotechnology.

[68] Mehwish N., Xu M., Zaeem M., Lee B.H. (2022). Mussel-Inspired Surface Functionalization of Porous Albumin Cryogels Supporting Synergistic Antibacterial/Antioxidant Activity and Bone-Like Apatite Formation. Gels.

[69] Kiran A.S.K., Kumar T.S., Sanghavi R., Doble M., Ramakrishna S. (2018). Antibacterial and Bioactive Surface Modifications of Titanium Implants by PCL/TiO_2_ Nanocomposite Coatings. Nanomaterials.

[70] Liu Z., Qin S., Wang W., Liu J., Liu D., Chen X., Li W., Mei B. (2022). Microstructure, Interface and Strengthening Mechanism of Ni-CNTs/AZ91 Magnesium Matrix Composites. Materials.

[71] Kúdela S., Koráb J., Štefánik P. (2022). Effect of Temperature on the Complex Modulus of Mg-Based Unidirectionally Aligned Carbon Fiber Composites. Materials.

[72] Wu Z., Song J., Zhang Y., Xue B., Wang S. (2022). Mechanism Analysis of Nanosecond Pulse Laser Etching of SiC_p_/Mg Composites. Materials.

[73] Lesz S., Karolus M., Gabryś A., Kremzer M. (2022). Influence of Milling Time on Phase Composition and Product Structure of Mg-Zn-Ca-Ag Alloys Obtained by Mechanical Synthesis. Materials.

[74] Chen J., Dai J., Qian J., Li W., Li R., Pang D., Wan G., Li P., Xu S. (2022). Influence of Surface Roughness on Biodegradability and Cytocompatibility of High-Purity Magnesium. Materials.

[75] Emelyanenko A.M., Domantovsky A.G., Kaminsky V.V., Pytskii I.S., Emelyanenko K.A., Boinovich L.B. (2021). The Mechanisms of Antibacterial Activity of Magnesium Alloys with Extreme Wettability. Materials.

[76] Helmholz H., Adejube B., Luthringer-Feyerabend B., Willumeit-Römer R. (2021). In Vitro Investigation on Degradable Mg-Based Biomaterial under the Impact of the Serum Glycoprotein Fetuin. Materials.

[77] Bakhtiari-Zamani H., Saebnoori E., Bakhsheshi-Rad H.R., Berto F. (2022). Corrosion and Wear Behavior of TiO_2_/TiN Duplex Coatings on Titanium by Plasma Electrolytic Oxidation and Gas Nitriding. Materials.

[78] Wang S.-H., Lee S.-P., Yang C.-W., Lo C.-M. (2021). Surface Modification of Biodegradable Mg-Based Scaffolds for Human Mesenchymal Stem Cell Proliferation and Osteogenic Differentiation. Materials.

[79] Yang C.-W., Wang G.-K. (2020). Effect of Hydrothermal (Sr)-Hydroxyapatite Coatings on the Corrosion Resistance and Mg^2+^ Ion Release to Enhance Osteoblastic Cell Responses of AZ91D Alloy. Materials.

[80] Mehwish N., Kausar A., Siddiq M. (2015). High-performance polyvinylidene fluoride/poly(styrene–butadiene–styrene)/functionalized MWCNTs-SCN-Ag nanocomposite membranes. Iran. Polym. J..

[81] Mehwish N., Kausar A., Siddiq M., Raheel M. (2015). Design and properties of polyvinylidene fluoride/poly (styrene-butadiene-styrene)/functionalized multi-walled carbon nanotube nanocomposite membranes. J. Plast. Film. Sheeting.

[82] Abazari S., Shamsipur A., Bakhsheshi-Rad H.R., Berto F. (2022). Functionalized carbon nanotube-encapsulated magnesium-based nanocomposites with outstanding mechanical and biological properties as load-bearing bone implants. Mater. Des..

[83] Shajahan, Mo Y., Kibria A.F., Kim M., Nahm K. (2004). High growth of SWNTs and MWNTs from C_2_H_2_ decomposition over Co–Mo/MgO catalysts. Carbon.

