# Correction: Amirzade-Iranaq et al. MWCNTs-TiO_2_ Incorporated-Mg Composites to Improve the Mechanical, Corrosion and Biological Characteristics for Use in Biomedical Fields. *Materials* 2023, *16*, 1919

**DOI:** 10.3390/ma18245617

**Published:** 2025-12-15

**Authors:** Mohammad Taher Amirzade-Iranaq, Mahdi Omidi, Hamid Reza Bakhsheshi-Rad, Abbas Saberi, Somayeh Abazari, Nadia Teymouri, Farid Naeimi, Claudia Sergi, Ahmad Fauzi Ismail, Safian Sharif, Filippo Berto

**Affiliations:** 1 Advanced Materials Research Center, Department of Materials Engineering, Najafabad Branch, Islamic Azad University, Najafabad, Iran; 2 Department of Materials and Metallurgical Engineering, Amirkabir University of Technology, Tehran, Iran; 3 Department of Chemical Engineering Materials Environment, Sapienza University of Rome, Eudossiana 18, 00184 Roma, Italy; 4 Advanced Membrane Technology Research Center (AMTEC), Universiti Teknologi Malaysia, Johor Bahru 81310, Johor, Malaysia; 5 Advanced Manufacturing Research Group, Faculty of Mechanical Engineering, Universiti Teknologi Malaysia, Johor Bahru 81310, Johor, Malaysia

In the original publication [1], there was a mistake in Figure 8 as published. The original Figure 8c,d have overlapped. The corrected Figure 8 appears below. The authors state that the scientific conclusions are unaffected. This correction was approved by the Academic Editor. The original publication has also been updated.

## Figures and Tables

**Figure 8:**
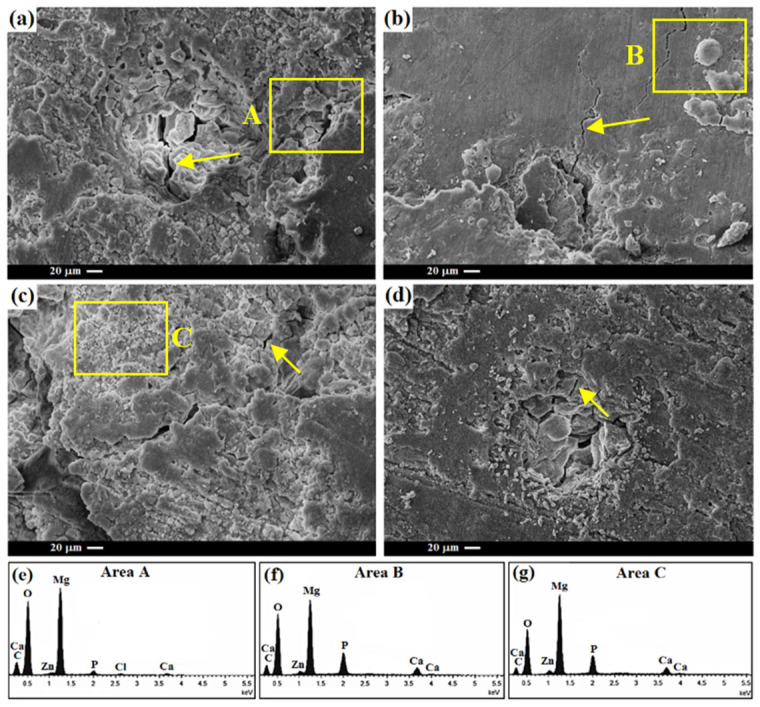
Surface morphology of the (**a**) TM0, (**b**) TM1, (**c**) TM2, (**d**) TM3 composites after 14 days of immersion in SBF and EDS analyses of (**e**) Area A, (**f**) Area B, and (**g**) Area C. Note: the arrow indicates the crack.
